# A modified tracheal transection approach for cervical esophageal lesion treatment: A report of 13 cases

**DOI:** 10.3389/fsurg.2022.1001488

**Published:** 2022-10-21

**Authors:** Yang Liu, Nan Huang, Wei Xu, Jie Liu, Changming An, Yiming Zhu, Shaoyan Liu, Zongmin Zhang

**Affiliations:** ^1^ Department of Head and Neck Surgical Oncology, National Cancer Center/National Clinical Research Center for Cancer/Cancer Hospital, Chinese Academy of Medical Sciences and Peking Union Medical College, Beijing, China; ^2^ Department of Head and Neck Surgery, Shandong Provincial ENT Hospital, Cheeloo College of Medicine, Shandong University, Jinan, China

**Keywords:** tracheal transection approach, cervical esophageal cancer, papillary thyroid cancer, preservation of laryngeal function, surgical technique

## Abstract

**Background:**

Surgical interventions for tumors in the cervical esophageal region are complicated and laryngeal function is frequently sacrificed. Therefore, we attempted the tracheal transection approach to resect the tumor while preserving laryngeal function.

**Methods:**

Three patients with papillary thyroid cancer (PTC), six with cervical esophageal cancer (CEC), and four with CEC mixed with thoracic esophageal cancer (TEC) were enrolled. The esophagus was exposed after the trachea was transected between the second and third tracheal rings. *CEC/TEC:* Resection of the esophagus or/and a portion of the hypopharynx with acceptable safety margins and repair with free jejunum or tubular stomach. *PTC*: Suture the small esophageal incision immediately after removing the tumor. The tracheal dissection was repaired with interrupted sutures throughout the entire layer after the esophageal lesion was resected. The status of the recurrent laryngeal nerve (RLN) determined whether a tracheotomy was necessary.

**Results:**

All 13 patients had effective esophageal lesion excision, with six of them requiring intraoperative tracheotomy. Postoperative complications included a tracheoesophageal fistula (one case, 7.7%), postoperative RLN paralysis (two cases, 15.4%), and aspiration (three cases, 23.1%). Except for two patients with distant metastases, there was no recurrence in the remaining patients after 5–92 months of follow-up.

**Conclusion:**

The tracheal transection approach, as a new surgical technique, can retain laryngeal function while ensuring appropriate exposure and satisfactory surgical resection. Before surgery, the feasibility of this approach must be carefully assessed. The RLN should be protected during the procedure. The operation is both safe and effective, with a wide range of applications.

## Introduction

Although the cervical esophagus starts superiorly at the esophageal entrance and extends down to the sternal notch, extending approximately 6–8 cm, it falls under the scope of head and neck surgery. Despite the fact that it only covers approximately 5 cm and is a small section of the esophagus, lesions such as primary cervical esophageal cancer (CEC) or invaded lesions by other cancers are not uncommon in this area. The presence of the trachea, which makes the operating space exceedingly tight, renders the care of carcinomas at this site particularly difficult. As a result, total pharyngeal, laryngeal, and esophageal resection is the gold standard for CEC treatment in the early stages (1). However, this extensive surgical resection frequently results in significant functional loss and has a negative impact on the patient's quality of life (2). For other carcinomas such as thyroid carcinoma that invade the esophagus beyond the midline, the presence of the trachea can also lead to blind areas of the visual field, and the tumor is prone to be incompletely removed. For this group of patients, we present herein an innovative operative technique to deal with cervical esophageal lesions *via* a modified tracheal transection approach to achieve better surgical results.

## Materials and methods

**Figure 1:**
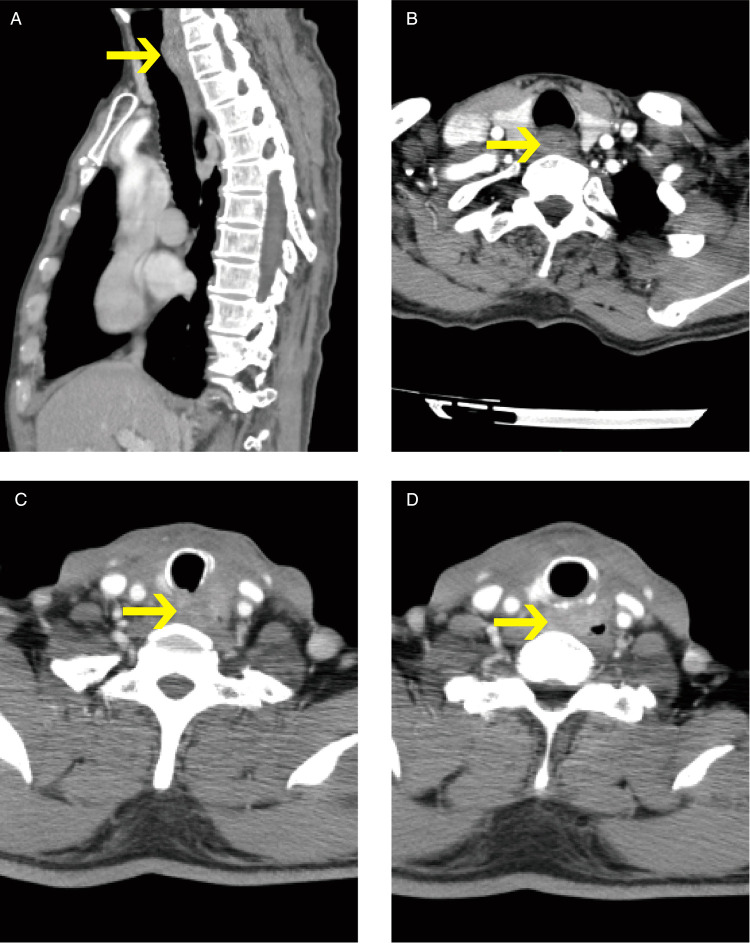
Ct findings of two patients (pictures in alphabetical order from top to bottom, left to right: ABCD. Pictures (**A,B**) show patient #1 CEC; Pictures (**C,D**) show patient #3 PTC. The arrows indicate the tumors.).

Thirteen patients treated in the Chinese Academy of Medical Sciences, the Peking Union Medical College Cancer Hospital, and Shandong ENT Hospital between October 2016 and September 2021 were enrolled. Before surgery, all patients were given complete information about the procedure and written informed consent was obtained. The patients' preoperative clinical characteristics are shown in Table 1 and CT findings of two patients are demonstrated in Figure 1.

**Table 1:** Clinical characteristics of the 13 patients.

Number	Age	Sex	Diagnosis	Preoperative therapy	cTNM Stage	Cancer Location (cm)
Case.1	69	Female	CEC	No	T2N1M0	16–20
Case.2	62	Male	CEC/TEC	No	T2N0M0	16–18/27–30
Case.3	56	Male	PTC	No	T4aN1bM1	/
Case.4	54	Male	PTC	No	T4aN1bM1	/
Case.5	18	Male	PTC	No	T4aN1bM0	/
Case.6	60	Male	CEC	Yes (R)	T2N0M0	17–20
Case.7	52	Male	CEC/TEC	No	T1N0M0	18–23/31–37
Case.8	62	Male	CEC	No	T1N1M0	20 cm
Case.9	56	Male	CEC	No	T4N0M0	18–22/27–32
Case.10	64	Male	CEC/TEC	No	T3N0M0	20 cm
Case.11	58	Male	CEC	No	T3N0M0	15–18
Case.12	66	Male	CEC/TEC	No	T2N0M0	15–17/25–28
Case.13	53	Male	CEC	YES (C)	T1N0M0	18–20

CEC, Cervical esophageal carcinoma; TC, Papillary Thyroid carcinoma; TEC, Thoracic esophageal carcinoma; R, radiotherapy; C, Chemotherapy.

In all 13 cases, there was only one female patient. Three patients were diagnosed with papillary thyroid cancer (PTC), six with CEC, and four with CEC and coexisting thoracic esophageal carcinoma (TEC). Patients were aged 18–69 years with an average age of 56.2 years. Only one patient with CEC accepted preoperative radiation therapy and one patient with CEC accepted preoperative chemotherapy. Because esophageal carcinoma reconstruction methods should be defined by the cancer's site, data for this section were also collected and are presented in Table 1. The AJCC Cancer Staging Manual, Eighth Edition, was used to establish all tumor staging (3).

### Surgical techniques

#### For thyroid cancer treatment

Preoperative examination of the potential amount of esophageal invasion and the selection of surgical approaches depending on the situation is required for all patients. Thyroid cancer patients with less significant esophageal invasion do not need a tracheal transection. However, for locally advanced thyroid cancer, preoperative imaging such as CT scan or MRI with contrast reveals that the tumor has invaded the esophagus beyond the dorsal midline of the esophagus. In this case, the conventional surgical approach may not be able to completely remove the tumor, and a tracheal transection approach may be applied according to the intraoperative evaluation. Because esophageal invasion is frequently associated with invasion of the recurrent laryngeal nerve (RLN), preoperative laryngoscopy is also required. All procedures were done under general anesthesia. A traditional collar neck incision was used to perform total thyroidectomy and neck dissection. The thyroid isthmus was initially transected, followed by lobectomy in the lobes with no nodules or smaller nodules, and if possible, with at least one RLN effectively intact. In our cohort, all three thyroid tumors were determined to have invaded at least one side of the RLN intraoperatively, which had to be sacrificed during surgery. After separating both sides of the thyroid gland from the trachea, the latter was fully exposed. Then the trachea was transected between the second and third tracheal rings.

While incising the trachea, the anesthesiologist drew the tracheal tube into the mouth in preparation for tracheal anastomosis and subsequent intubation. The prepared sterile tube was inserted into the broken end of the trachea and given to the anesthesiologist to manage ventilation. The two segments of the trachea were stretched separately up and down so that the cervical esophagus and tumor were fully exposed. Care was taken to avoid excessive stretching of the RLN. Because all three patients exhibited partial esophageal invasion, the esophageal defect could be directly sutured after *en bloc* excision of the partially invaded esophagus, the remnant thyroid lobe, and the invaded RLN. RLN signals were detected *via* intraoperative nerve monitoring (IONM). Two patients underwent tracheotomy because their RLN signals were diminished, while the third patient had an end-to-end anastomosis of the trachea.

#### For esophageal cancer treatment

Some of the initial esophagectomy and neck dissection techniques were similar to the thyroid cancer surgical procedure detailed earlier. The tracheal transection approach can be performed only on patients in whom the feasibility of laryngeal preservation has been confirmed preoperatively. The trachea was transected between the second and third tracheal rings, and the posterior wall of the trachea was carefully checked to see if it had been invaded. The pharyngeal cavity was opened by cutting the pharyngeal constrictor muscle from the side, and the upper boundary of the esophageal cancer lesion was examined, and we checked for any hypopharyngeal invasion. After that, we dissected and protected both RLNs before performing cervical esophagectomy.

**Figure 2:**
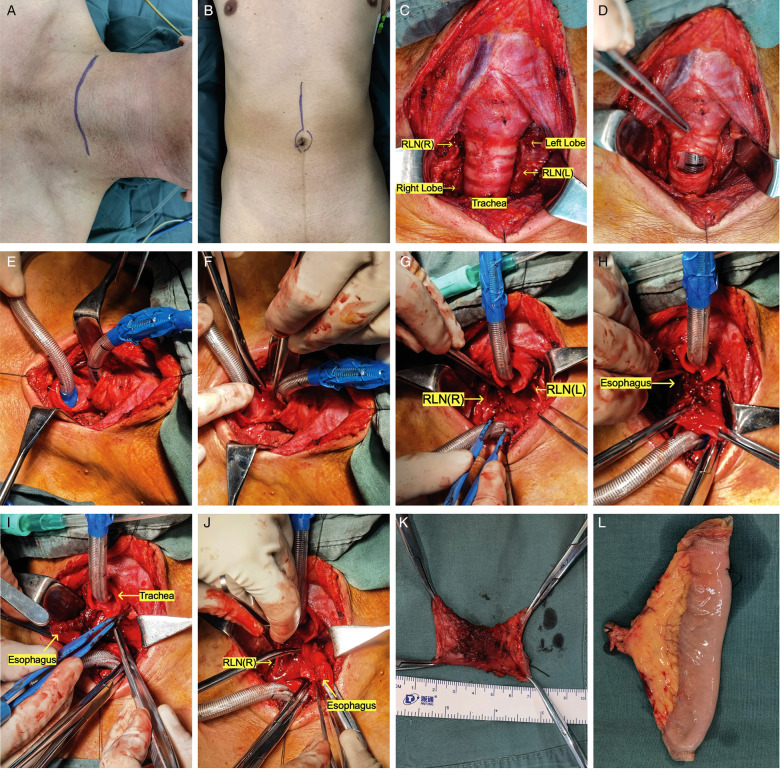

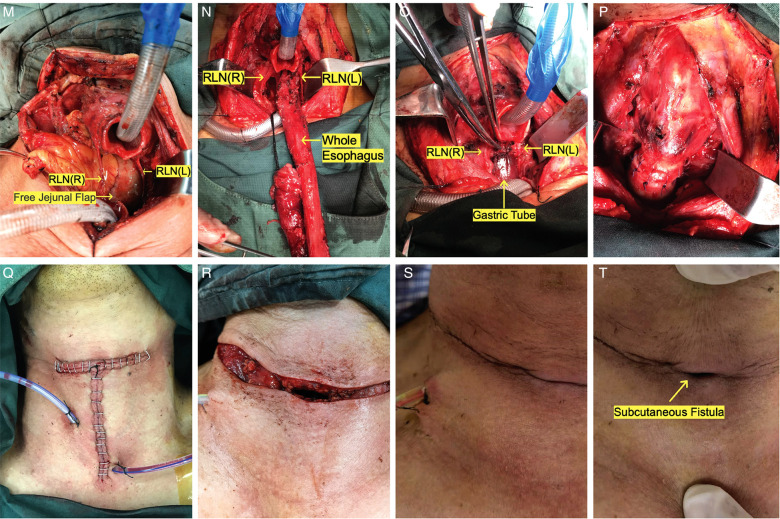
Surgery pictures (the photos of two patients undergoing surgery were chosen to demonstrate the various surgical repairs and tracheal management techniques. Pictures A-M, R-T show patient #11; Pictures N-Q show patient #2). Pictures in alphabetical order from top to bottom, left to right: ABCDEFGHIGKLMNOPQRST. (**A**) Neck incision (a collar incision or a T-shaped incision). (**B**) Abdominal incision (free jejunum). (**C**) Dissect the isthmus of the thyroid and draw both lobes outward to show the trachea and bilateral RLN on the inside. (**D**) The trachea was transected between the second and third tracheal rings. (**E**) Replace the tracheal intubation. (**F**) Cutting the posterior wall of the trachea. (**G**) To figure out where the upper limit of cervical esophageal cancer is and whether the larynx can be preserved. (**H**) Separate the tracheoesophageal space, taking care not to overstretch the recurrent laryngeal nerves on either side. (**I**) The esophagus is incised from the tumor's bottom border (ensuring adequate safety borders), and the prevertebral fascia is checked for invasion. (**J**) The esophagus is incised from the tumor's upper border (ensuring adequate safety borders). (**K**) Cervical esophageal specimens that have been excised. (**L**) Free jejunal flap (repair of the cervical esophageal defect). (**M**) The free jejunum is sutured to the defective upper and lower esophageal anastomoses in preparation for micro-anastomosis of arterial and venous vessels. (**N**) In patients with CEC and TEC, the procedure necessitates the resection of the entire esophagus, which usually necessitates the assistance of a thoracic surgeon to free the lower esophagus so that the entire esophagus can be pulled to the neck. (**O**) The gastric tube is retracted to the neck for anastomosis. It is important to avoid over-distention of the RLN throughout this procedure. (**P**) The tracheal wall can be sutured all around in individuals with no invasion of the recurrent laryngeal nerve and no excessive intraoperative strain. (**Q**) Following the placement of the drainage tube, the incision was immediately closed (no tracheostomy was performed). (**R**) If surgery reveals an invasion of the recurrent laryngeal nerve, or if the recurrent laryngeal nerve is too stretched to determine whether the signal is intact, a subcutaneous fistula (suturing the anterior wall of the trachea to the subcutaneous tissue) is feasible. (**S**) Third postoperative day. No tracheal tube was inserted. The fistula will progressively close over time if there is no significant asphyxia. (**T**) If hypoxia is present, the fistula can be vented by slightly tilting the head back or manually pulling the fistula opening to the sides.

Total esophagectomy should be performed on patients with CEC and coexisting TEC. Depending on the tumor's location and invasive range, a free jejunal flap or gastric tube was used. Finally, we sutured the broken end of the trachea and, depending on the situation, conducted a tracheotomy (if necessary), fistulated through the anterior wall of the trachea, and placed an indwelling tracheal cannula. If the status of the RLN cannot be verified, a subcutaneous tracheostomy can also be performed without placing a tracheal tube first. The fistula can be vented by tilting the head back slightly or manually pulling the fistula opening to the sides if hypoxia is present (Figure 2).

### Follow-up plan

All patients were discharged from the hospital and given a follow-up plan. Patients were examined every 3 months for the first 2 years and then every 6 months thereafter. Neck ultrasound, enhanced CT of the neck and chest, electronic laryngoscopy, and gastroscopy are routine post-operative examinations. When clinical signs or imaging tests point to the possibility of distant metastases, bone scintigraphy, magnetic resonance imaging (MRI), or positron emission computed tomography (PET) will be conducted, depending on the situation.

## Results

Esophageal defects in all three thyroid cancer cases were sutured directly. Free jejunal flaps were chosen in half of the CEC or CEC combined TEC cases, and gastric pull-up was performed in the other half. Tracheotomy was required in six (46.2%) patients. Postoperative complications included tracheoesophageal fistula (one case, 7.7%), postoperative RLN paralysis (two cases, 15.4%), and aspiration (three cases, 23.1%). There was no postoperative bleeding or tracheostenosis (Table 2). All patients resumed oral intake on postoperative day 7–10, with the exception of case 2, who suffered from postoperative tracheoesophageal fistula leading to a short-term nasogastric tube feeding, which is reversed by a tracheal stent implantation. The patient resumed oral intake afterwards.

**Table 2:** Information related to surgery and postoperative complications in 13 patients.

Number	Date of surgery	Repairing Method	Tracheotomy	SND	Pathology	pTNM Stage	Anastomotic Fistula	RLNP	Bleeding	TS	Aspiration
Case.1	2/20/2017	free jejunal flap	No	Yes	SCC	T3N1M0	No	No	No	No	Yes
Case.2	9/1/2017	gastric tube	No	Yes	SCC	T3N0M0	Yes^a^	No	No	No	No
Case.3	4/28/2017	sutured directly	Yes	Yes	PTC	T4aN0M1	No	Yes (pre-op)	No	No	No
Case.4	1/3/2017	sutured directly	No	Yes	PTC	T4aN1aM1	No	Yes (pre-op)	No	No	No
Case.5	6/20/2014	sutured directly	Yes	Yes	PTC	T4aN1bM0	No	Yes (pre-op)	No	No	Yes
Case.6	10/10/2016	free jejunal flap	No	Yes	SCC	T2N0M0	No	No	No	No	No
Case.7	9/12/2017	gastric pull up	No	Yes	SCC	TisN0M0	No	Yes (post-op)	No	No	No
Case.8	11/2/2017	free jejunal flap	Yes	Yes	SCC	T1bN1M0	No	No	No	No	Yes
Case.9	1/14/2018	gastric pull up	No	Yes	SCC	T4bN1M0	No	No	No	No	No
Case.10	1/24/2018	gastric pull up	Yes	Yes	SCC	T4bN0M0	No	Yes (post-op)	No	No	No
Case.11	6/20/2018	free jejunal flap	Yes	Yes	SCC	T3N0M0	No	No	No	No	No
Case.12	9/16/2021	gastric pull up	Yes	Yes	SCC	T1bN0M0	No	No	No	No	No
Case.13	8/26/2021	free jejunal flap	No	Yes	SCC	T0N0M0	No	No	No	No	No

SND, selective neck dissection; RLNP, recurrent laryngeal nerve paralysis; Pre-op, preoperative; Post-op, postoperative; TS, tracheostenosis; SCC, squamous cell carcinoma; PTC, papillary thyroid carcinoma.

^a^ Tracheoesophageal fistula.

Two patients with thyroid cancer had postoperative radioactive iodine^131^ treatment, and five patients with CEC, one with thyroid cancer, and one with CEC combined with TEC received postoperative radiotherapy. The remaining four patients had no postoperative treatment and were only followed-up. This group of patients was followed-up for a total of 5–92 months. Except for two patients with definite bilateral RLN palsy who required postoperative arytenoid cartilage resection or posterior vocal cord dissection before the tracheal cannula was removed, the latter was withdrawn between 3 days and 1 month in all patients. One patient with CEC and TEC died of myocardial infarction 26 months after surgery, another patient with PTC died of brain metastasis combined with renal failure 23 months after surgery, and another patient with CEC died of bone metastasis 27 months after surgery. The remaining patients had no tumor recurrence (Table 3).

**Table 3:** Follow-up information of the 13 patients.

Number	Postoperative Treatment	Follow-up (month)	Results
Case.1	Radiotherapy	27	osseous metastasis, dead
Case.2	No	49	recurrence-free survival
Case.3	I^131^	23	brain metastases and renal failure, dead
Case.4	I^131^	61	recurrence-free survival
Case.5	Radiotherapy	92	recurrence-free survival
Case.6	Radiotherapy	58	recurrence-free survival
Case.7	No	52	recurrence-free survival
Case.8	Radiotherapy	50	recurrence-free survival
Case.9	Radiotherapy	48	recurrence-free survival
Case.10	Radiotherapy	26	myocardial infarction, dead
Case.11	Radiotherapy	43	recurrence-free survival
Case.12	No	5	recurrence-free survival
Case.13	No	6	recurrence-free survival

I^131^, radioiodine^131^.

## Discussion

Because of its anatomical location, the involvement of the trachea often limits visual field exposure and complicates the surgical technique, regardless of whether it is a primary cancer of the cervical esophagus or other cancers invading the esophagus. Previous surgical treatment of cervical esophageal cancer has frequently jeopardized laryngeal function. However, the larynx and trachea are frequently uninvolved in these patients, and the larynx is removed simply to fully expose the hypopharynx and esophageal entrance behind the larynx. The patient's ability to talk is lost after a total laryngectomy, which increases the burden of care and has a substantial influence on the patient's quality of life. Similarly, tracheal blockage often leads to incomplete surgical resection and is associated with a risk of recurrence in patients with thyroid cancer that significantly invades the esophagus, especially those with recurrent thyroid cancer after many surgical interventions. As a result, a surgical approach that preserves the larynx while eliminating the lesion is urgently needed. We found that the modified tracheal transection approach is a superior surgical technique, which is simple for surgeons who have mastered the tracheal sleeve resection method, a commonly used method in patients with tracheal malignancy and thyroid cancer invading the trachea (4–6). In addition, direct suturing after tracheal transection is safer than tracheal sleeve resection, which is more prone to complications due to increased tension in the suture.

As one of the most prevalent endocrine malignancies (7), well-differentiated thyroid cancer is associated with an approximate 13% rate of local invasion (8), while esophageal invasion accounts for approximately 21% of invasive thyroid cancer cases (9, 10). Although the overall prognosis for thyroid cancer is favorable, locally-advanced thyroid cancer frequently causes serious complications such as airway obstruction, bleeding, or dysphagia, and survival for patients with and without the involvement of the aerodigestive tract differs significantly (10, 11), making surgery a crucial part of the treatment. Different scholars have different opinions on surgical resection for this group of patients. Some researchers feel that only shave excision can be conducted for patients with tumors that are difficult to remove cleanly by visual inspection, and that other postoperative treatments can be adjuvant (12). However, based on our experience and certain published findings (13, 14), we believe that for locally invasive thyroid cancer, we should also opt for radical excision to achieve a better prognosis. Therefore, a tracheal transection technique can better achieve tumor radicalization in papillary thyroid cancer that has invaded the esophagus beyond the midline. Furthermore, because anastomosis after tracheal transection does not increase anastomotic tension, this procedure does not significantly increase the risk of anastomotic fistula and is safe and dependable in comparison to the anastomotic dehiscence complication rate of 4%–25% reported in the literature (4, 15, 16). Due to the insufficient sample size, we could not detect any anastomotic fistula issues in our patients. We will extend the sample for further statistical analysis.

Locally advanced thyroid cancer or central metastatic lymph nodes can typically infiltrate the esophagus, particularly on the left side, and are frequently accompanied by invasion of the RLN or trachea. In addition to regular preoperative examinations, ultrasound, CT, MRI, laryngoscopy, and gastroscopy, ultrasound endoscopy if necessary, and additional tracheoscopy if there is tracheal invasion, because this group of patients requires a thorough preoperative assessment. Because the mucosa of the esophagus is a tough barrier to penetrate, differentiated thyroid cancer seldom penetrates the mucosal layer of the esophagus and is predominantly located outside the lumen; therefore, the tumor can be considered to be excised *en bloc* from the submucosal layer of the esophagus, and the myocutaneous layer can be sutured *in situ*, with no further reconstruction required because the closure is rarely under tension. Empirically, a feeding tube can aid in the identification of the esophagus during surgery. Likewise, in our group, there were no major abnormalities in all three papillary thyroid cancer instances, and all (3, 100%) of the defects were primarily closed.

It is sometimes difficult to heal *in situ* after the removal of tumors that penetrate the entire layer, which might result in esophageal stenosis. To widen the esophageal lumen and ensure the quality of swallowing after surgery, tissue transfer may be necessary (17, 18). Fascial or fasciocutaneous flaps, myocutaneous pedicled flaps, or jejunal, gastric transfers may be good options, depending on the deficits and the surgeon's inclination. During the follow-up period, only one patient in our study died due to renal failure. The remaining two patients had no recurrence at the time of the final follow-up. No patient experienced evident complications such as dysphagia and bleeding. This evidence shows that the process is both safe and effective.

Cervical esophageal cancer is uncommon, accounting for approximately 2%–10% of all esophageal malignancies (19, 20). The treatment of cervical esophageal cancer is more complicated, and a comprehensive treatment plan that combines surgery, radiotherapy, chemotherapy, and immunotherapy is becoming increasingly identified (21–24). However, the vast number of surrounding vital organs and the intricacy of the surgical stages, which have a greater impact on the patient's quality of life following surgery, make surgery for CEC a significant clinical challenge.

Squamous cell carcinoma, which accounts for more than 90% of all pathological types of cervical esophageal cancer (25, 26), requires a safer resection margin than papillary thyroid carcinoma surgery. Surgical treatment of cervical esophageal cancer in the past, particularly those affecting the esophagus's entrance and the hypopharynx, frequently sacrificed laryngeal function. Total laryngeal resection, total esophageal resection, and a permanent tracheostomy were commonly used in traditional operations (1), which resulted in language loss and pneumonia due to inadequate airway management. The larynx and trachea are frequently uninvolved in these patients, and the larynx is removed simply to fully expose the hypopharynx and esophageal entrance behind its body. As a result, an increasing number of surgeons are choosing laryngeal preservation surgery for patients who do not have considerable laryngeal and tracheal invasion. It was recently discovered that the mortality rate of complete or partial larynx-preserving operations was not significantly lower than that of larynx-non-preserving operations, and more inspiringly that larynx-preserving operations were not associated with a higher incidence of complications such as anastomotic leakage, pneumonia, graft necrosis, or infection (27–29). As a result of the improved quality of life, patients are more likely to accept this operation. Thus, a thorough preoperative evaluation is carried out to determine whether the larynx can be preserved, and there is a higher requirement for a surgical treatment that preserves the larynx while removing the lesion.

The tracheal transection method could solve all of the above concerns. The key methods to achieve a favorable surgical result are to expose the surgical field and improve the height of the gastropharyngeal anastomosis as much as possible during surgery. It was discovered that the tracheal transection method has a distinct advantage in that it can maximize operation field views, allowing for a clear exposure of the cervical esophagus. The involvement of the larynx and trachea can be reliably assessed with an adequate surgical field. The cervical esophagus can be resected and sutured under direct eyesight. Although the anastomosis height may be increased as much as possible, the suture might be more carefully placed to avoid the occurrence of a fistula. The remaining nine patients with CEC and patients with CEC combined with TEC did not develop anastomotic fistulas, with the exception of one patient with CEC combined with TEC (1, 10%) who developed a postoperative tracheoesophageal fistula. However, this is definitely lower than the 16.36% rate of anastomotic fistulas after surgery described in the literature (30).

Different from thyroid cancer surgery, the demand of deficiency repairing is essential for esophageal cancer surgery. For patient with CEC, jejunal repair is an option, whereas gastric substitution esophageal repair is essential for patients with CEC combined with TEC. There are also subtle differences in the strategy to tracheal transection for these two patient categories. As thoracic esophageal cancer is not within the area of treatment provided by our department, we will not discuss it here. In our experience, the stomach cannot be directly anastomosed to the hypopharynx in patients undergoing gastric substitution repair in order to prevent acid reflux-induced chemical pneumonia as a complication, the incidence of which have been documented in the medical literature to be approximately 11.1%–28.9% (2, 31, 32). The anastomosis is typically around 1 cm below the esophageal inlet, so the position of the tracheal transection is chosen to avoid the anastomosis as much as possible. According to the patient’s condition, the tracheal transection can be conducted between the first and second tracheal rings. In comparation, for patients with CEC, particularly those with esophageal cancer invading the hypopharynx, jejunal repair is required. Since the gastric reflux is less likely as a complication of jejunal repair, the tracheal transection position can be selected mainly based on the surgical condition, and typically we select space between the second and the third tracheal rings for transection.

It should be noted that because cervical esophageal cancer is so proximal to the RLN, preoperative electronic laryngoscopy, or at the very least, indirect laryngoscopy, should be frequently evaluated in the case of vocal cord paralysis. According to the literature, the rate of laryngeal recurrent nerve injury in individuals undergoing only surgery for cervical esophageal cancer might be up to 12.96%–28.3% (29, 30). The trachea and larynx are frequently moved upward along the tracheal stump to expose the posterior cervical esophagus after tracheal transection to provide a broader operation field. Our experience is to meticulously expose and dissect the RLN on both sides before pulling. Dissection of both sides of the RLN is usually started after disconnecting the isthmus of the thyroid gland. There is some relaxation after dissection, since the RLN is usually not tight. When drawing the tracheal stump and larynx forward, it is important to be gentle, pay attention to the nerve's tension, and avoid damaging the RLN by excessive pulling. If possible, IONM can also be used.

To minimize bilateral RLN palsy affecting respiration, additional attention should be devoted to safeguarding the contralateral RLN in patients who have had one RLN palsy prior to surgery. The procedure must be carried out with caution. In our study, 20% patients experienced transient RLN palsy on one side after surgery, as well as hoarseness, which could be related to the dissection and retraction of the RLN during surgery; this incidence is slightly lower than the 28.3% reported in the literature (29). If at least one unilateral RLN signal is normal and the trachea is not considerably invaded, tracheotomy may not be necessary even after tracheal transection. Fortunately, because the RLN's integrity was intact, most of the traction-induced RLN palsy was temporary, and the two patients' bilateral vocal cord motions were normal 6 months after the operation. If the IONM is not used in the operation and the surgeon determines that the traction on the nerve during the procedure is severe, the posterior wall of the trachea can be sutured and a tracheotomy or tracheostomy performed on the anterior wall for safety.

For patients undergoing prophylactic tracheotomy, according to our experience, a subcutaneous fistula can be chosen, in which the free end of the tracheostomy stoma is sutured and fixed to the subcutaneous tissue of anterior neck while leaving the skin layer unsutured, and the tracheal tube is not placed after the operation. The patient is then observed for any obvious asphyxia, if any, the skin covering the tracheocutaneous stoma can be pulled up to expose the fistula and a tracheal intubation *via* the stoma can be conducted immediately, if none, the fistula opening will close spontaneously. The tracheal cannula can be removed for the closure of fistula as soon as possible if there are no clear signs of asphyxia after the obturator is inserted. Because the literature indicates the tracheal cannula should be removed as soon as possible in order to prevent the adverse effect of positive pressure ventilation on sutures (33, 34). Similarly, if the trachea is found to be invaded during the procedure, it can be sutured directly after the sleeve resection. A tracheostomy can be performed in the first stage and repaired in the second stage if the tracheal defect is large. Only one tracheotomy was performed in patients who had a definite loss of RLN signals during surgery. Because IONM was not employed in all of the patients, four (40%) patients had to undergo tracheotomy during surgery. Except for three patients who had postoperative aspiration (30%), one patient developed a postoperative anastomotic fistula (10%). In this group, significant problems like hemorrhage and tracheal stenosis did not occur. Even though some patients had complications such as aspiration, anastomotic fistulas, or RLN paralysis, early detection and treatment may result in a better outcome and are associated with the overall prognosis (21, 25, 30). In our study, 10 patients were enrolled, with one patient dying 26 months after surgery from myocardial infarction and another dying 27 months later after surgery from osseous metastases. Overall, for patients with CEC or CEC combined with TEC, this surgical intervention is often safe and successful.

## Conclusion

The tracheal transection approach described herein is a novel surgical technique that can preserve laryngeal function while ensuring adequate exposure and satisfactory surgical resection for cervical esophageal tumors that do not involve the post-cricoid region and differentiated thyroid carcinoma that invades the esophagus beyond the midline. The extent of tumor invasion determines the appropriate approach; typically, the lower cervical border of the tumor does not surpass the thoracic entrance, the upper border does not exceed 2 cm above the esophageal entrance, and the hypopharyngeal lesion does not involve the posterior cricoid region. The laryngeal nerve should be protected during the procedure. Aspiration, recurrent laryngeal nerve paralysis, and tracheoesophageal fistula are the most common postoperative complications. The rate of complications with this approach is not higher than that associated with the conventional technique, and this surgical approach is safe, dependable, and can be applied in clinical practice.

## Data Availability

The raw data supporting the conclusions of this article will be made available by the authors, without undue reservation.
